# Inventory Replenishment and Transshipment for Distributed Autologous Cell Therapy Manufacturing Networks

**DOI:** 10.1002/bit.70316

**Published:** 2026-07-26

**Authors:** Zhaowei Li, Chelsea C. White, Kan Wang, Ben Wang

**Affiliations:** ^1^ H. Milton Stewart School of Industrial and Systems Engineering Georgia Institute of Technology Atlanta Georgia USA; ^2^ Georgia Tech Manufacturing Institute Georgia Institute of Technology Atlanta Georgia USA; ^3^ School of Materials Science and Engineering Georgia Institute of Technology Atlanta Georgia USA

**Keywords:** agent‐based simulation, autologous cell therapy, cell manufacturing, Markov decision processes, point‐of‐care, supply chain

## Abstract

Autologous cell therapy (AuCT) manufacturing networks must balance two competing objectives: reducing cost and improving patient access to time‐sensitive therapy. This study compares three AuCT manufacturing network designs: a centralized network with a single manufacturing facility, a coordinated point‐of‐care (POC) network in which POCs can coordinate and transship specimens and consumables when needed, and an independent POC network in which each POC operates separately. Minimizing the time from specimen harvest to therapy infusion is used as an operational proxy for patient benefit. The analysis considers reagent replenishment, specimen and reagent transshipment, patient deterioration while waiting for therapy manufacturing to begin or during the therapy manufacturing process, and bioreactor capacity planning under a high patient service‐level requirement. Using data based on a commercial‐style AuCT network with one manufacturing site, 20 geographically distributed POCs, and two consumable suppliers, the results show that the coordinated POC network can achieve total cost close to that of the centralized network while maintaining turnaround time close to that of the independent POC network. These findings provide decision‐support insight for commercialization and industrialization of AuCT manufacturing by quantifying when coordinated distributed manufacturing may offer a practical compromise between the economic efficiency of centralized production and the responsiveness of local manufacturing.

## 1 Introduction

Autologous cell therapy (AuCT) has emerged as a promising treatment platform for several severe diseases and has attracted growing attention as cell and gene therapy (CGT) programs move from clinical development toward commercialization (Rosenberg and Restifo 2015; June et al. 2018). Unlike conventional large‐batch biopharmaceutical production, AuCT manufacturing is patient‐specific, time‐sensitive, and operationally coupled to specimen collection, manufacturing‐slot availability, consumable positioning, chain‐of‐identity requirements, and therapy return to the treatment site. Prior work on commercial T‐cell and CAR‐T manufacturing emphasizes that scale‐up is constrained not only by biological process performance, but also by manufacturing capacity, logistics coordination, quality‐system consistency, and the ability to deliver patient‐specific products reliably across multiple sites (Kaiser et al. 2015; Levine et al. 2017; Elverum and Whitman 2020). These characteristics make manufacturing network design a central question for industrialization, because decisions regarding facility configuration, capacity deployment, and logistics coordination directly affect both cost and patient access (Teng et al. 2014; Wang et al. n.d).

A major strategic choice in AuCT manufacturing is whether therapy production should be centralized in one manufacturing facility or distributed to points of care (POCs). Centralized manufacturing can benefit from economies of scale, consolidated expertise, and coordinated resource planning. In contrast, local manufacturing at POCs can reduce transportation burden and shorten the time from specimen harvest to therapy infusion, a metric that is directly relevant to patient benefit and operational responsiveness. However, decentralized CGT manufacturing also raises practical questions regarding automation, comparability, governance, and resource utilization across sites (Harrison et al. 2018). Between these two extremes lies a coordinated distributed design in which multiple POCs manufacture locally but cooperate through cross‐site coordination and transshipment. This type of network is especially interesting from a commercialization perspective because it may preserve much of the responsiveness of local manufacturing while mitigating the cost penalties associated with fully independent operations.

The practical importance of this question is amplified by the distinctive operational features of AuCT supply chains. Manufacturing demand is uncertain and geographically dispersed, patients may deteriorate while waiting for treatment, key consumables must be positioned before use, and reusable bioreactor capacity must be installed in a way that satisfies stringent service‐level requirements. Consequently, network design decisions cannot be evaluated solely in terms of cost minimization or solely in terms of manufacturing speed. Rather, they must be assessed in terms of the trade‐off between total expected cost and the time from specimen harvest to therapy infusion, while accounting for the underlying operational realities of patient‐specific manufacturing.

Previous work has shown that distributed and coordinated POC configurations can substantially improve turnaround time relative to a centralized network and can reduce the cost gap between centralized and local manufacturing under favorable demand and service‐level conditions (Wang et al. n.d). Related work has also highlighted the role of capacity planning in decentralized AuCT manufacturing networks (Li and White 2022). More broadly, prior studies have emphasized that manufacturing configuration, supply chain strategy, and operational coordination are critical to the commercial feasibility of regenerative medicine and cell‐based therapies (Teng et al. 2014; Harrison et al. 2018; Elverum and Whitman 2020). The literature therefore motivates a decision‐support framework that connects manufacturing‐network structure to cost, service level, and patient‐facing responsiveness rather than treating the manufacturing model as a purely mathematical abstraction. However, there remains a need for an analytic framework that jointly captures reagent replenishment, patient deterioration, specimen and reagent transshipment, and bioreactor capacity planning in a way that is directly interpretable for AuCT manufacturing decisions.

This study addresses that need by developing an infinite‐horizon, discounted‐cost Markov decision process (MDP) formulation for three AuCT manufacturing network designs: a centralized network, a coordinated POC network, and an independent POC network. The model incorporates reagent replenishment, specimen and reagent transshipment, patient deterioration while waiting for therapy manufacturing to begin or during the therapy manufacturing process, and bioreactor capacity planning under a high service‐level requirement. In the numerical study, this service‐level requirement is defined as a 0.95 probability that manufacturing can begin immediately upon arrival of the patient specimen at the facility where manufacturing is scheduled to occur. In addition to the MDP formulation, the study develops a practical reagent replenishment heuristic for the coordinated POC network, a myopic transshipment heuristic, and a capacity planning procedure for determining the number of bioreactors needed to satisfy the target service level.

The goal of the paper is not only to provide a mathematically structured comparison of alternative AuCT network designs, but also to generate insights that are useful for commercialization and industrialization decisions in the CGT field. In particular, the analysis is intended to help clarify when coordinated distributed manufacturing may offer a desirable compromise between the lower cost of centralized production and the faster turnaround associated with local manufacturing. Such insights are relevant to manufacturers, treatment centers, and supply‐chain planners who must decide how to configure multi‐site AuCT manufacturing systems under stringent patient service requirements.

## 2 Model and Assumptions

This section presents the operational model used to determine reagent replenishment and transshipment decisions under the initial assumption that idle bioreactor capacity is sufficient to allow manufacturing to begin immediately once a specimen is available. The capacity‐limiting assumption is relaxed later in the paper in order to determine the minimum number of bioreactors required to satisfy the target patient service level.

The decision process can be interpreted as a repeating weekly operating cycle for an AuCT manufacturing network with N manufacturing facilities (when N=1, the network is centralized). At the beginning of each decision epoch, each facility observes the current queue of waiting specimens and its available reagent inventory. Reagent replenishment decisions are made first, after which replenishment arrivals, new patient specimens, and patient‐deterioration events are realized. For the coordinated POC network, specimen and reagent transshipment decisions are then made to rebalance the system before manufacturing starts. Manufacturing can begin only when a specimen, a unit of reagent, and an idle bioreactor are simultaneously available. The resulting post‐production state becomes the starting state for the next decision epoch. Under this interpretation, the sequence of events in each period is as follows:

1. A reagent replenishment decision is made for each manufacturing facility, based on the number of specimens currently waiting for manufacturing to begin and the number of units of reagent currently in inventory for all manufacturing facilities in the network.
2. The reagent replenishment orders arrive, new specimens arrive, and patient transitions from eligible to ineligible take place.
3. Based on these additional data, specimen and reagent transshipment decisions are determined for the coordinated POC network (transshipments do not occur for the centralized or independent POC networks). Due to current FDA regulations, bioreactors are assumed not to be transshipped.
4. Once transshipments are completed, therapy manufacturing begins. Therapy manufacturing can only begin for a patient if, besides the patient's specimen, there is an available idle bioreactor (initially always assumed to be available) and an available unit of reagent.
5. For patients whose therapy manufacturing begins, the patient's specimen, the concomitant unit of reagent, and the idle bioreactor are each removed from their respective inventories, which determines the number of specimens waiting for manufacturing to begin, the number of units of reagent in inventory for all facilities in the network, and the state of the bioreactors for all facilities in the network at the next decision epoch.

### 2.1 Nomenclature

Assume s(t,n) is the number of specimens waiting for manufacturing to start at time t at facility n,r(t,n) is the number of units of reagents in inventory at time t at facility n, and let x(t,n)=(s(t,n),r(t,n)). Let a(t,n) be the number of reagents ordered for facility n at time t, a decision determined for each n, based on x(t)={x(t,n)}, and let a(t)={a(t,n)}.

Let the random variable d(t,n) represent the number of specimens that arrive at facility n at time t, and let the random variable θ(s) be the number of patients who become ineligible out of s eligible patients. The realizations of both d(t,n) and θ(s(t,n)) are revealed after the reagent replenishment decisions are made. Once a patient becomes ineligible, the patient is assumed to depart the system and never return.

Therefore, just before transshipment decisions are made, the number of specimens and units of reagents at facility n are

(1) $$\tilde{s}\left(t,n\right)=s\left(t,n\right)-\theta \left(s\left(t,n\right)\right)+d\left(t,n\right)$$

(2) $$\tilde{r}\left(t,n\right)=r\left(t,n\right)+a\left(t,n\right)$$

Transshipment decisions are then determined, based on knowledge of $\left\{\tilde{s}\left(t,n\right),\tilde{r}\left(t,n\right)\right\}$. Let $y_{s}\left(m,n\right)\left(t\right)$ ($y_{r}\left(m,n\right)\left(t\right)$) be the number of specimens (units of reagents) transshipped from facility m to facility n between t and $t+1,y_{s}\left(t\right)=\left\{y_{s}\left(m,n\right)\left(t\right)\right\},y_{r}\left(t\right)=\left\{y_{r}\left(m,n\right)\left(t\right)\right\}$, and $y\left(t\right)=\left(y_{s}\left(t\right),y_{r}\left(t\right)\right)$. The single period transshipment cost SPTC(t) accrued between t and t+1 is

(3) $${\mathrm{SPTC}}_{t}=\sum_{m,n}c_{s}\left(m,n\right)y_{s}\left(m,n\right)\left(t\right)+c_{r}\left(m,n\right)y_{r}\left(m,n\right)\left(t\right)$$

where $c_{s}\left(m,n\right)$ ($c_{r}\left(m,n\right)$) is the cost of transshipping one specimen (unit of reagent) from facility m to facility n. Following transshipment, the resulting number of specimens and reagents at facility n become

(4) $$\hat{s}\left(t,n\right)=\tilde{s}\left(t,n\right)+\sum_{m}\left(y_{s}\left(m,n\right)\left(t\right)-y_{s}\left(n,m\right)\left(t\right)\right)\geq 0$$

(5) $$\hat{r}\left(t,n\right)=\tilde{r}\left(t,n\right)+\sum_{m}(y_{r}\left(m,n\right)\left(t\right)-y_{r}\left(n,m\right)\left(t\right))\geq 0$$

The number of specimens that then begin manufacturing at facility n is $\hat{m}\left(t,n\right)=\min\left(\hat{s}\left(t,n\right),\hat{r}\left(t,n\right)\right)$. Thus, at the next decision epoch, the number of specimens waiting for therapy manufacturing to begin and the number of unused units of reagents are

(6) $$s\left(t+1,n\right)=\tilde{s}\left(t,n\right)-\hat{m}\left(t,n\right)$$

(7) $$r\left(t+1,n\right)=\tilde{r}\left(t,n\right)-\hat{m}\left(t,n\right)$$

The single‐period total cost accrued between epochs t and t+1 is

(8) $$\begin{array}{rcl} c\left(x\left(t\right),a\left(t\right),y\left(t\right)\right) & = & c_{R}\sum_{n}a\left(t,n\right)\\ & + & p\sum_{n}s\left(t+1,n\right)\\ & + & h\sum_{n}r\left(t+1,n\right)\\ & + & \mathrm{SPTC}\left(t\right) \end{array}$$

where $c_{R}$ is the cost of a unit of reagent, p is the penalty of delaying the start of manufacturing for a patient until the next epoch, and h is the holding cost for a single period for a single unit of reagent. The value of p can be inferred from the chance constraint

(9) $$P\left(\sum_{n}s\left(t+1,n\right)\leq \sum_{n}r\left(t+1,n\right)\right)\geq \left(1-\alpha \right)$$

based on a technique presented in Li (2021). The value of p is considerably greater than any of the other costs, which would typically be the case for small values of α, a reasonable assumption in this domain.

The problem criterion is given by:

(10) $$E\left\{\sum_{t=1}^{\infty }\beta^{t-1}c(x\left(t\right),a\left(t\right),y\left(t\right))\right\},$$

assuming the discount factor β<1. The problem objective is to determine a reagent replenishment policy and a transshipment policy that minimizes the criterion.

Following a standard solution procedure for the MDP (Puterman 1994), let the operator H be defined as:

(11) $$H\left(v\left(x,t\right)\right)=\min_{a\left(t\right),y\left(t\right)}E[c\left(x\left(t\right),a\left(t\right),y\left(t\right)\right)+\beta v\left(x\left(t+1\right)\right)].$$

Since the minimum of a(t) and the minimum of y(t) are based on different data, the optimality equation can be rewritten as:

(12) $$H\left(v\left(x\left(t\right)\right)\right)=\min_{a\left(t\right)}\min_{y\left(t\right)}\left\{E\left[p\sum_{n}\tilde{s}\left(t,n\right)\right.\right.+h\sum_{n}\tilde{r}\left(t,n\right)+c_{R}\sum_{n}a\left(t,n\right)+\min\left(\mathrm{SPTC}-\left(p+h\right)\sum_{n}\hat{m}\left(t,n\right)\right)+\beta v\left(x\left(t+1\right)\right)]\},$$

where the inner minimization is subject to the inequalities:

(13) $$\hat{s}\left(t,n\right)=\tilde{s}\left(t,n\right)+\sum_{m}(y_{s}\left(m,n\right)\left(t\right)-y_{s}\left(n,m\right)\left(t\right))\geq 0$$

(14) $$\hat{r}\left(t,n\right)=\tilde{r}\left(t,n\right)+\sum_{m}(y_{r}\left(m,n\right)\left(t\right)-y_{r}\left(n,m\right)\left(t\right))\geq 0$$

and the outer minimization is such that a(t,n)≥0 for all n. Standard results for the MDP (Puterman 1994) show that there exists a unique fixed‐point $v^{*}$ for the operator H, that is, $v^{*}=Hv^{*}$, this fixed point is the value of the criterion minimized over all replenishment and transshipment policies, and the values of $a\left(t,n\right),y_{s}\left(m,n\right)\left(t\right)$, and $y_{r}\left(m,n\right)\left(t\right)$ that cause the minima to be obtained are optimal decisions.

### 2.2 Lower Bound

Note that the minimum number of reagents required satisfies the inequality:

(15) $$\min\left(\sum_{n}\tilde{s}\left(t,n\right),\sum_{n}\tilde{r}\left(t,n\right)\right)\geq \sum_{n}\hat{m}\left(t,n\right)$$

and that

(16) $$\sum_{n}\hat{s}\left(t,n\right)=\sum_{n}\tilde{s}\left(t,n\right)$$

(17) $$\sum_{n}\hat{r}\left(t,n\right)=\sum_{n}\tilde{r}\left(t,n\right).$$

Thus, the operator for the lower bound can be expressed as:

(18) $$[Hv]\left(x(t)\right)\geq [H^{L}v]\left(x(t)\right)=\min E\left\{p\sum_{n}\tilde{s}(t,n)+h\sum_{n}\tilde{r}(t,n)\right.+c_{R}\sum_{n}a(t,n)+\min[\mathrm{SPTC}-\left(p+h\right)\times \min\left(\sum_{n}\tilde{s}(t,n),\sum_{n}\tilde{r}(t,n)\right)+\beta v\left(x(t+1)\right)]\}.$$

Given the properties of operators H and $H^{L}$, the fixed point of $H^{L}$ serves as a lower bound for the fixed point of H. There is always a transshipment decision that can transform the inequality in (13) to an equality. Since p is significantly larger than any transshipment costs, decisions will aim for equality in (13).

Assuming the summation of θ(s(t,n)) across all n equals $\theta (\sum_{n}s(t,n))$, a condition met by the binomial distribution, it follows that $H^{L}v$ depends solely on $\sum_{n}\tilde{s}(t,n)$ and $\sum_{n}\tilde{r}(t,n)$, rendering transshipping unnecessary and setting SPTC to 0. Consequently:

(19) $$[H^{L}v](x\left(t\right))=\min E\{c_{R}\sum_{n}a\left(t,n\right)+p{(\sum_{n}s\left(t,n\right)+\phi \left(t\right)-(\sum_{n}r\left(t,n\right)+\sum_{n}a\left(t,n\right)}^{+}+h{(\sum_{n}r\left(t,n\right)+\sum_{n}a\left(t,n\right)-(\sum_{n}s\left(t,n\right)+\phi \left(t\right)}^{+}+\beta v(x\left(t+1\right))\}.$$

Define $\phi \left(\xi \right)=\sum_{n}d\left(t,n\right)+\theta \left(\xi \right)$ and $\phi \left(t\right)=\phi (\sum_{n}s\left(t,n\right))$. It follows from results in Puterman (1994), the fixed point of $H^{L}$ adheres to an optimal (myopic) policy.

The reagent ordering policy for facility n at time t is given by:

(20) $$a\left(t,n\right)=s\left(t,n\right)-r\left(t,n\right)+F_{\phi \left(t-1\right)}^{-1}\left(\rho \right),$$

where $\rho =\frac{p}{p+h}$, and $F_{\phi \left(t\right)}^{-1}\left(\rho \right)$ represents the inverse cumulative distribution function of the random variable ϕ(t) for the given parameter ρ, equating to an optimal solution for the centralized network.

The expectation of the total cost, minimized by the policy, is:

(21) $$\min E\left\{c_{R}\sum_{n}a\left(t,n\right)+p\left(\sum_{n}s\left(t,n\right)\right)\right.-p\left(\sum_{n}r\left(t,n\right)\right)+\sum_{n}a\left(t,n\right)+\left\|\sum_{n}r\left(t,n\right)-\sum_{n}s\left(t,n\right)\right\|+\phi (t)\}=E\left\{c_{R}\sum_{n}a\left(t,n\right)+p{\left(\phi \left(t\right)-F_{\phi \left(t\right)}^{-1}\left(\rho \right)\right)}^{+}\right.+\left.h{\left(F_{\phi \left(t\right)}^{-1}\left(\rho \right)-\phi \left(t\right)\right)}^{+}\right\}\},$$

which is minimized when θ(ξ)=0 for all ξ≥0. Consequently, for θ(ξ)=0 for all ξ≥0 and all x(t):

(22) $$\mathrm{LB}\leq v^{*}\left(x\left(t\right)\right)=\frac{1}{1-\beta }E\{c_{R}\sum_{n}a\left(t,n\right)+p{(\phi \left(t\right)-F_{\phi \left(t\right)}^{-1}\left(\rho \right)}^{+}+h{(F_{\phi \left(t\right)}^{-1}\left(\rho \right)-\phi \left(t\right)}^{+}\}$$

### 2.3 Upper Bound

Note the relationship between the operator Hv and the upper bound $H^{U}v$:

(23) $$\left[Hv](x\left(t\right))\leq [H^{U}v](x\left(t\right))=\min E\left\{c_{R}\sum_{n}a\left(t,n\right)+p\sum_{n}\tilde{s}\left(t,n\right)\right.+h\sum_{n}\tilde{r}\left(t,n\right)-\left(p+h\right)\sum_{n}\hat{m}\left(t,n\right)+\beta v(x\left(t+1\right))\right\}$$

where $y_{s}(m,n)=y_{r}(m,n)=0$ for all m and n is sub‐optimal for the inner minimization. Hence, $\tilde{s}(t,n)=\hat{s}(t,n)$ and $\tilde{r}(t,n)=\hat{r}(t,n)$ for all n.

The fixed point of $H^{U}v$ can be expressed as the sum over all n of the fixed points of operators in the form:

(24) $$\min_{n}E\;\{c_{R}a\left(t,n\right)+p\tilde{s}\left(t,n\right)+h\tilde{r}\left(t,n\right)-\left(p+h\right)\min(\tilde{s}\left(t,n\right),\tilde{r}\left(t,n\right))+\beta v(s\left(t+1,n\right),r\left(t+1,n\right))\}$$

which effectively describes a network of N totally independent manufacturing facilities, akin to the solution of the independent POC network.

(25) $$E\;\{c_{R}a\left(t,n\right)+p\tilde{s}\left(t,n\right)+h\tilde{r}\left(t,n\right)-\left(p+h\right)\min(\tilde{s}\left(t,n\right),\tilde{r}\left(t,n\right))+\beta v(s\left(t+1,n\right),r\left(t+1,n\right))\}=E\{c_{R}a\left(t,n\right)+p{\left(\tilde{s}\left(t,n\right)-\min(\tilde{s}\left(t,n\right),\tilde{r}\left(t,n\right))\right)}^{+}+h{\left(\tilde{r}\left(t,n\right)-\min(\tilde{s}\left(t,n\right),\tilde{r}\left(t,n\right))\right)}^{+}+\beta v(s\left(t+1,n\right),r\left(t+1,n\right))\},$$

Define the function:

(26) ϕ(t,n)=d(t,n)−θ(s(t,n)),

and the optimal (myopic) policy:

(27) $$a\left(t,n\right)=s\left(t,n\right)-r\left(t,n\right)+F_{\phi \left(t,n\right)}^{-1}\left(\rho \right),$$

where $\rho =\frac{p}{p+h}$. For all x(t), the upper bound UB is:

(28) $$\begin{array}{ccc} v^{*}\left(x\left(t\right)\right) & \leq & UB=\sum_{n}\frac{E\{c_{R}a\left(t,n\right)}{1-\beta }\\ & + & \frac{p\left(\phi \left(t,n\right)-F_{\phi \left(t,n\right)}^{-1}\left(\rho \right)\right)}{1-\beta }\\ & + & \frac{h\left(F_{\phi \left(t,n\right)}^{-1}\left(\rho \right)-\phi \left(t,n\right)\right)}{1-\beta } \end{array}$$

The difference in the total amount of reagent ordered between the upper bound and the lower bound is given by:

(29) $$\sum_{n}F_{\phi \left(t,n\right)}^{-1}\left(\rho \right)-F_{\phi \left(t-1\right)}^{-1}\left(\rho \right)\geq 0.$$

As the penalty parameter p increases (or equivalently, as the patient service level increases), the difference UB−LB narrows, converging to 0 in the limit.

## 3 The Reagent Replenishment Heuristic

This section presents a practical replenishment rule for the coordinated POC network. Operationally, the replenishment decision must be made before new specimen arrivals, patient‐deterioration events, and transshipment actions are observed. The relevant planning question is therefore the following: how much reagent should be positioned across the coordinated network at the beginning of the period so that the network remains adequately protected against short‐term demand while avoiding the excessive local safety stock implied by a fully independent POC design?

The proposed rule addresses this question by selecting a coordinated‐network reagent level that lies between two analytically meaningful benchmarks. The first benchmark is the centralized design, which pools uncertainty across the entire network and therefore requires the smallest aggregate protection stock. The second benchmark is the independent POC design, which protects each site locally and therefore requires a larger aggregate reagent level. The coordinated POC network should, in principle, operate between these two limits: it can exploit a portion of the pooling benefit through network coordination, but it does not have the full physical consolidation of the centralized design.

Let

(30) $$a\left(t,n\right)=s\left(t,n\right)-r\left(t,n\right)+\frac{F_{\phi \left(t,n\right)}^{-1}\left(\rho \right)}{\sum_{m}F_{\phi \left(t,m\right)}^{-1}\left(\rho \right)}\xi$$

where ξ is a design parameter that satisfies

(31) $$F_{\phi \left(t\right)}^{-1}\left(\rho \right)\leq \xi \leq \sum_{m}F_{\phi \left(t,m\right)}^{-1}\left(\rho \right).$$

For notational convenience, define the lower and upper benchmark values

$$\xi_{L}=F_{\phi \left(t\right)}^{-1}\left(\rho \right),\xi_{U}=\sum_{m}F_{\phi \left(t,m\right)}^{-1}\left(\rho \right).$$

The parameter ξ therefore represents the total amount of reagent positioned across the coordinated network before transshipment decisions are made. When $\xi =\xi_{U}$, the replenishment decision coincides with the independent POC benchmark; when $\xi =\xi_{L}$, the total reagent level matches the centralized benchmark. In managerial terms, ξ can be interpreted as a network‐level protection parameter: smaller values emphasize centralized pooling logic, whereas larger values emphasize local autonomy and site‐level protection.

The replenishment rule is intentionally myopic. The decision a(t,n) is determined before transshipment is observed and is not conditioned directly on the realized transshipment‐cost structure. This simplification is useful in the present setting because it yields a replenishment procedure that is interpretable, computationally tractable, and compatible with routine operational planning. The computational procedure has two components. Algorithm 1 performs an iterative search over feasible candidate values of ξ, and Algorithm 2 evaluates the total expected‐cost consequence of a given candidate value and provides the finite‐difference slope estimate used by the search routine.

**Table:** 

**ALGORITHM 1 |** Iterative search for the coordinated‐network reagent level ξ.
**Require:** Feasible interval $\left[\xi_{L},\xi_{U}\right]$ defined by (31), initial candidate $\xi^{\left(0\right)}$, step size η, maximum number of iterations K, convergence tolerance ϵ, discount factor β, service‐level parameter α, finite‐difference increment δ>0
**Ensure:** Selected coordinated‐network reagent level $\xi^{⋆}$
1: Set k←0 and project the initial candidate onto the feasible interval:
$\xi^{\left(0\right)}\leftarrow \min\left\{\xi_{U},\max\left\{\xi_{L},\xi^{\left(0\right)}\right\}\right\}$
2: Evaluate the total expected cost associated with the initial candidate:
$J^{\left(0\right)}\;\leftarrow$ evaluatecandidate $\left(\xi^{\left(0\right)},\xi_{L},\xi_{U},\beta ,\alpha ,\delta \right)$
3: **while** k<K **do**
4: Estimate the local slope of the criterion with respect to $\xi^{\left(k\right)}$:
$g^{\left(k\right)}\;\leftarrow$ gradientestimate $\left(\xi^{\left(k\right)},\xi_{L},\xi_{U},\beta ,\alpha ,\delta \right)$
5: Generate an updated candidate and enforce feasibility:
$\tilde{\xi }\leftarrow \min\left\{\xi_{U},\max\left\{\xi_{L},\xi^{\left(k\right)}-\eta g^{\left(k\right)}\right\}\right\}$
6: Evaluate the total expected cost at the updated candidate:
$\tilde{J}\;\leftarrow$ evaluatecandidate $\left(\tilde{\xi },\xi_{L},\xi_{U},\beta ,\alpha ,\delta \right)$
7: **if** $|\tilde{J}-J^{\left(k\right)}|<\epsilon$ **then**
8: **break**
9: **end if**
10: Accept the updated candidate:
$\xi^{\left(k+1\right)}\leftarrow \tilde{\xi },J^{\left(k+1\right)}\leftarrow \tilde{J}$
11: Set k←k+1
12: **end while**
13: **return** $\xi^{⋆}=\xi^{\left(k\right)}$

**Table:** 

**ALGORITHM 2 |** Evaluation of a candidate coordinated‐network reagent level ξ.
**Require:** Candidate reagent level ξ, feasible interval $\left[\xi_{L},\xi_{U}\right]$, discount factor β, service‐level parameter α, finite‐difference increment δ>0
**Ensure:** Total expected cost J(ξ) and gradient estimate g(ξ)
1: Determine the penalty parameter implied by the service‐level requirement α under candidate ξ
2: Evaluate the total expected cost of the coordinated POC network under candidate ξ:
J(ξ)← total discounted cost induced by the replenishment rule associated with ξ
3: Form two neighboring feasible candidates for a finite‐difference calculation:
$\xi^{+}\leftarrow \min\left\{\xi_{U},\xi +\delta \right\},\xi^{-}\leftarrow \max\left\{\xi_{L},\xi -\delta \right\}$
4: Evaluate the total expected cost at the neighboring candidates:
$J\left(\xi^{+}\right)$ and $J\left(\xi^{-}\right)$
5: Compute the finite‐difference gradient estimate:
$g\left(\xi \right)\leftarrow \frac{J\left(\xi^{+}\right)-J\left(\xi^{-}\right)}{\xi^{+}-\xi^{-}}$
6: **return** J(ξ) and g(ξ)

Taken together, Algorithms 1 and 2 provide a practical search‐and‐evaluate framework for selecting the coordinated‐network reagent level. Algorithm 1 defines the feasible search logic, stopping rule, and update mechanism for the candidate parameter, whereas Algorithm 2 maps each candidate value to the total expected‐cost criterion used for comparison and supplies the finite‐difference slope information required by the search. The resulting procedure is not intended to produce a closed‐form optimal solution to the full stochastic control problem; rather, it yields a transparent and implementable approximation that places the coordinated POC design between the analytically motivated centralized and independent POC limits.

## 4 The Transshipment Heuristic

The transshipment model determines one‐period specimen and reagent movements for the coordinated POC network after arrivals and patient‐status changes have been observed but before manufacturing begins. Its role is therefore different from that of the replenishment heuristic. Whereas replenishment determines how much inventory should be positioned in anticipation of uncertainty, transshipment determines how realized specimen and reagent imbalances should be corrected after the period's uncertainty has been observed. In this sense, the transshipment procedure performs short‐term operational rebalancing.

The objective of the transshipment rule is to start manufacturing for as many patients as possible at minimum transshipment cost. This objective is particularly important in the coordinated POC setting because the value of coordination arises precisely from the ability to move material and work across sites rather than forcing each POC to absorb its own local variability. The transshipment model therefore uses the realized specimen counts, reagent counts, and available bioreactor capacity at each site to determine where specimens and reagents should be moved during the current decision epoch.

The transshipment heuristic is represented by an integer program, summarized in Algorithm 3, that minimizes the one‐period movement cost while satisfying

(32) $$\min\left(\sum_{n}s\left(t,n\right),\sum_{n}r\left(t,n\right)\right)=\sum_{n}m\left(t,n\right),$$

subject to

(33) $$\hat{s}\left(t,n\right)=\tilde{s}\left(t,n\right)+\sum_{m}(y_{s}\left(m,n\right)-y_{s}\left(n,m\right))\geq 0,$$

(34) $$\hat{r}\left(t,n\right)=\tilde{r}\left(t,n\right)+\sum_{m}(y_{r}\left(m,n\right)-y_{r}\left(n,m\right))\geq 0.$$

These constraints ensure that post‐transshipment specimen and reagent balances remain feasible at every facility. The decision variables $y_{s}\left(m,n,t\right)$ and $y_{r}\left(m,n,t\right)$ therefore have a direct operational interpretation: they represent the specimen and reagent flows assigned between facilities during the current decision epoch.

As with the replenishment rule, the transshipment procedure is intentionally myopic. It does not optimize the downstream value‐function term βv(x(t+1)) explicitly; instead, it focuses on the immediate operational objective of restoring balance across the network so that available specimens, reagents, and capacity can be matched effectively in the current period. This choice is deliberate because it preserves transparency, computational tractability, and direct operational interpretability.

**Table:** 

**ALGORITHM 3 |** Myopic transshipment heuristic for the coordinated POC network.
**Require:** Realized specimen counts $\tilde{s}\left(t,n\right)$, realized reagent counts $\tilde{r}\left(t,n\right)$, idle bioreactor counts b(t,n,0), and transshipment costs $c_{s}\left(m,n\right),c_{r}\left(m,n\right)$
**Ensure:** One‐period specimen and reagent transshipment plans $y_{s}\left(m,n,t\right),y_{r}\left(m,n,t\right)$
1: Formulate an integer program with decision variables $y_{s}\left(m,n,t\right)$ and $y_{r}\left(m,n,t\right)$
2: Minimize the one‐period transshipment cost:
$\sum_{m,n}c_{s}\left(m,n\right)\;y_{s}\left(m,n,t\right)+\sum_{m,n}c_{r}\left(m,n\right)\;y_{r}\left(m,n,t\right)$
3: Subject to the post‐transshipment balance constraints in (33) and (34)
4: Impose manufacturing‐feasibility constraints so that the resulting post‐transshipment state supports $\hat{m}\left(t,n\right)$ therapy starts at each facility
5: Solve the integer program to obtain the minimum‐cost one‐period transshipment plan
6: Update the post‐transshipment specimen and reagent levels, $\hat{s}\left(t,n\right)$ and $\hat{r}\left(t,n\right)$, together with the implied manufacturing starts $\hat{m}\left(t,n\right)$
7: **return** $y_{s}\left(m,n,t\right),y_{r}\left(m,n,t\right)$

Algorithm 3 should therefore be interpreted as the operational bridge between replenishment and manufacturing. Once replenishment has positioned reagent inventory across the network and the period's uncertainty has been realized, the transshipment step reallocates resources so that the coordinated POC network can exploit its cross‐site flexibility. This is the mechanism through which the coordinated network recovers much of the responsiveness of distributed manufacturing without fully incurring the cost penalty of independent local operation.

## 5 Algorithm for Manufacturing Capacity Planning

The preceding analysis assumes that bioreactor capacity is sufficiently large that an idle bioreactor is always available when manufacturing is ready to begin. This assumption is useful for isolating the operating decisions associated with reagent replenishment and transshipment, but it is not sufficient for strategic network design. In practice, bioreactors are fixed capital assets installed at specific facilities, and the number installed at each location directly affects both patient service performance and total cost. Capacity planning is therefore a strategic design problem layered on top of the operational policies introduced above.

The purpose of this section is to determine how many bioreactors should be installed in the centralized network and at each POC in the independent POC network when the replenishment and transshipment rules are treated as fixed operational policies. The resulting procedure does not re‐optimize all operational decisions simultaneously. Instead, it evaluates the total discounted‐cost consequence of alternative installed‐capacity plans and searches for a low‐cost assignment that still satisfies the target patient service level. This decomposition is attractive in the present setting because it separates the longer‐term capital‐investment question from the shorter‐term operating decisions.

The criterion is expanded to incorporate the one‐time cost of bioreactor purchases,

(35) $$c_{B}\cdot \sum_{n}b\left(1,n,0\right)+E\left[\sum_{t=1}^{\infty }\beta^{t-1}c\left(x_{t},a_{t},y_{t}\right)\right],$$

where $c_{B}$ is the expense of purchasing a single bioreactor and the discount factor again satisfies β<1. The problem objective is to determine the number of bioreactors in the network that minimizes the expanded criterion, assuming the heuristic replenishment and transshipment policies developed above are deployed while ensuring the patient service level is satisfied.

The specimen and reagent process dynamics are as defined in Equations (1, 2, and (4), (5), (6), (7)); the bioreactor process dynamics are defined as follows. Let b(t,n,τ) be the number of therapies/bioreactors τ epochs from completion at facility n and let the vector b(t,n)=(b(t,n,0),b(t,n,1),…,b(t,n,T−1)), where b(t,n,0) is the number of idle bioreactors at facility n and T is the number of epochs (e.g., weeks) required for cell therapy manufacturing completion (e.g., T=3). Including the state of the bioreactors, define x(t,n)=(s(t,n),b(t,n),r(t,n)) and $\hat{m}\left(t,n\right)=\min\left(\hat{s}\left(t,n\right),b\left(t,n,0\right),\hat{r}\left(t,n\right)\right)$. The dynamics of the bioreactors are

(36) $$b\left(t+1,n,0\right)=b\left(t,n,0\right)-m\left(t,n\right)+b\left(t,n,1\right)+\theta b\left(t,n,T-1\right)+\sum_{\tau =2}^{T-1}\theta b\left(t,n,\tau \right)$$

(37) $$\begin{array}{ccc} b\left(t+1,n,\tau \right) & = & b\left(t,n,\tau +1\right)-\theta b\left(t,n,\tau +1\right),\\ & & \tau =1,\ldots ,T-2 \end{array}$$

(38) $$b\left(t+1,n,T-1\right)=\hat{m}\left(t,n\right)-\theta \hat{m}\left(t,n\right).$$

These dynamics capture the assumption that if a patient with therapy in production becomes ineligible, the therapy being manufactured is removed from the bioreactor and discarded and the bioreactor is sanitized and returned to the idle‐bioreactor set by the next decision epoch.

It follows that

(39) $$\begin{array}{ccc} \min & & \left(\sum_{n}\tilde{s}\left(t,n\right),\sum_{n}b\left(t,n,0\right),\sum_{n}\tilde{r}\left(t,n\right)\right)\\ & = & \min\left(\sum_{n}\hat{s}\left(t,n\right),\sum_{n}b\left(t,n,0\right),\sum_{n}\hat{r}\left(t,n\right)\right)\\ & \geq & \sum_{n}\hat{m}\left(t,n\right). \end{array}$$

It is shown in Li and White (2022) that there exists {b(1,1,0),…,b(1,N,0)} such that there is an optimal expected total discounted operation cost and that a global minimum can be iteratively searched using a coordinate‐descent procedure. The intuition behind this approach is straightforward: each coordinate corresponds to the installed bioreactor count at one facility, and the algorithm repeatedly asks whether adding or removing a small number of bioreactors at that facility improves the overall objective while preserving the service‐level requirement.

More specifically, at iteration k, the coordinate‐descent search selects a coordinate, chooses a search step size $\delta_{k}$, and evaluates the total discounted cost associated with a local capacity decrease, no change, and a local capacity increase at the selected facility. In practical terms, this means that the algorithm compares the three candidate plans

$$\begin{array}{ccc} & & x\left(1\right)\left(b\left(1,1,0\right),\ldots ,b\left(1,i_{k},0\right)-\delta_{k},\ldots ,b\left(1,N,0\right)\right),\\ & & x\left(1\right)\left(b\left(1,1,0\right),\ldots ,b\left(1,i_{k},0\right),\ldots ,b\left(1,N,0\right)\right),\\ & & x\left(1\right)\left(b\left(1,1,0\right),\ldots ,b\left(1,i_{k},0\right)+\delta_{k},\ldots ,b\left(1,N,0\right)\right), \end{array}$$

subject to feasibility with respect to the patient service‐level requirement. The current installed‐capacity value at the selected facility is then updated to the best of these candidates according to the total discounted‐cost criterion.

Algorithm 4 summarizes the resulting coordinate‐descent search. The output of the procedure is a bioreactor installation plan that can be interpreted directly by a network planner: it specifies how many bioreactors should be installed at the centralized site or at each POC, depending on the network design under evaluation. The procedure terminates when no single‐coordinate adjustment yields a meaningful improvement in the objective, thereby producing a locally improved capacity plan that balances capital cost, operating cost, and service performance.

**Table:** 

**ALGORITHM 4 |** Coordinate‐descent procedure for bioreactor capacity planning.
**Require:** Initial installed‐capacity vector $b^{\left(0\right)}=\left(b_{1,0}^{\left(1\right)},\ldots ,b_{1,0}^{\left(N\right)}\right)$, nonincreasing step‐size sequence $\left\{\delta_{k}\right\}$, maximum number of iterations K, and target patient service level
**Ensure:** Locally improved installed‐capacity vector $b^{⋆}$
1: Set k←0 and evaluate the total discounted cost associated with $b^{\left(0\right)}$
2: **while** k<K **do**
3: Select a coordinate $i_{k}\in \left\{1,\ldots ,N\right\}$ corresponding to facility $i_{k}$
4: Construct the candidate installed‐capacity plans
$b^{-}=b^{\left(k\right)}-\delta_{k}e_{i_{k}},b^{0}=b^{\left(k\right)}$, and $b^{+}=b^{\left(k\right)}+\delta_{k}e_{i_{k}}$
5: For each feasible candidate plan, evaluate the total discounted cost under the replenishment and transshipment rules defined earlier
6: Select the lowest‐cost candidate that satisfies the patient service‐level requirement
7: **if** a candidate capacity adjustment improves the objective **then**
8: Set $b^{\left(k+1\right)}$ equal to the selected candidate plan
9: **else**
10: Set $b^{\left(k+1\right)}\leftarrow b^{\left(k\right)}$
11: **end if**
12: **if** no improving single‐coordinate adjustment exists and $\delta_{k}=1$ **then**
13: **break**
14: **end if**
15: Update the step size if required and set k←k+1
16: **end while**
17: **return** $b^{⋆}=b^{\left(k\right)}$

## 6 Numerical Results

This section compares the three network designs under a baseline numerical case study constructed to reflect a commercial‐style AuCT manufacturing network. The analysis focuses on two outcomes that are central to commercialization and industrialization: the annual cost required to operate and equip the network, and the time from specimen harvest to therapy infusion. The first outcome reflects economic scalability for manufacturers and supply‐chain planners, whereas the second provides an operational proxy for patient benefit and treatment‐center responsiveness. Cost inputs are based on the equipment and cost assumptions listed in Appendix B, and the resulting insights are compared with those reported in Wang et al. (n.d).

### 6.1 Assumptions

For the baseline numerical study, the centralized manufacturing facility is assumed to be located in El Segundo, California, and the 20 POCs are the treatment sites shown in Figure 1 (see Appendix A for location details and the numbered POC key). Demand at each POC is modeled as an independent Poisson random variable with an average annual demand of 250 patients. As noted in Wang et al. (n.d), the numerical comparison can be sensitive to changes in average annual demand; a broader sensitivity analysis with respect to demand is therefore an important topic for future work. The patient transition probability is set to 0.05. For each network design, a high patient service level is defined as a 0.95 probability that manufacturing can begin immediately upon arrival of the patient specimen at the facility where manufacturing is scheduled to occur; the penalty‐function approach in Li (2021) is used to enforce the corresponding chance constraint. The discount factor is fixed at 0.9 throughout.

- The number of bioreactors needed is 325 for the centralized network and 21 for each POC for the independent POC network, for a total of 420, which represents a 29% increase for the total number of bioreactors for the independent POC network, compared to the centralized network. For the coordinated POC network, 325 bioreactors satisfy the patient service level; however, 328 bioreactors minimize total cost, representing less than a 1% increase in the total number of bioreactors needed, compared to the centralized network.
- The reagent base stock level for the centralized network is 117 and nine for each POC for the independent POC network, for a total of 180, representing a 54% increase for the sum of the base stock levels for the independent POC network, compared to the centralized network. For the coordinated POC network, a total base stock level of 117 satisfies the patient service level; however, a base stock level of six per POC for a total sum of 120 minimizes total cost, representing a 2.6% increase in the total base stock level for the coordinated POC network, compared to the centralized network.

**FIGURE 1:**
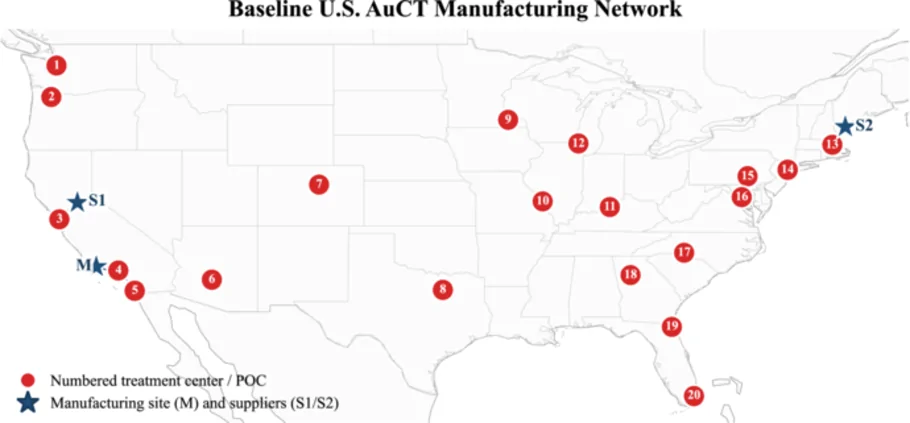
Geographic configuration of the baseline AuCT manufacturing network used in the numerical study. Numbered red markers denote the 20 treatment centers/POCs listed in Appendix A; blue star markers denote the centralized manufacturing site (M) and the two supplier locations (S1 and S2) used in the transportation‐cost calculations. Transportation distances and times derived from this commercial‐style U.S. network are used in the cost and turnaround‐time analyses.

### 6.2 Simulation Output Analysis

Because the AnyLogic model is stochastic, repeated runs of the same network configuration produce different annual cost outcomes due to random patient arrivals, patient deterioration events, and transshipment‐related operational dynamics. The simulation replication, rather than the individual patient, is treated as the experimental unit because observations within a run are coupled through shared resource states, inventory levels, and transshipment decisions. Each network configuration was therefore evaluated using R=50 independent simulation replications with distinct random seeds.

Let $C_{rjk}$ denote the annual cost observed in replication r∈{1,…,R}, for network configuration j, and cost component k. The primary estimand for each bar in Figure 2 is the expected annual cost component, $\mu_{jk}=E\left[C_{rjk}\right]$. The corresponding simulation estimator is the replication‐based sample mean

$${\hat{\mu }}_{jk}=\frac{1}{R}\sum_{r=1}^{R}C_{rjk}.$$

**FIGURE 2:**
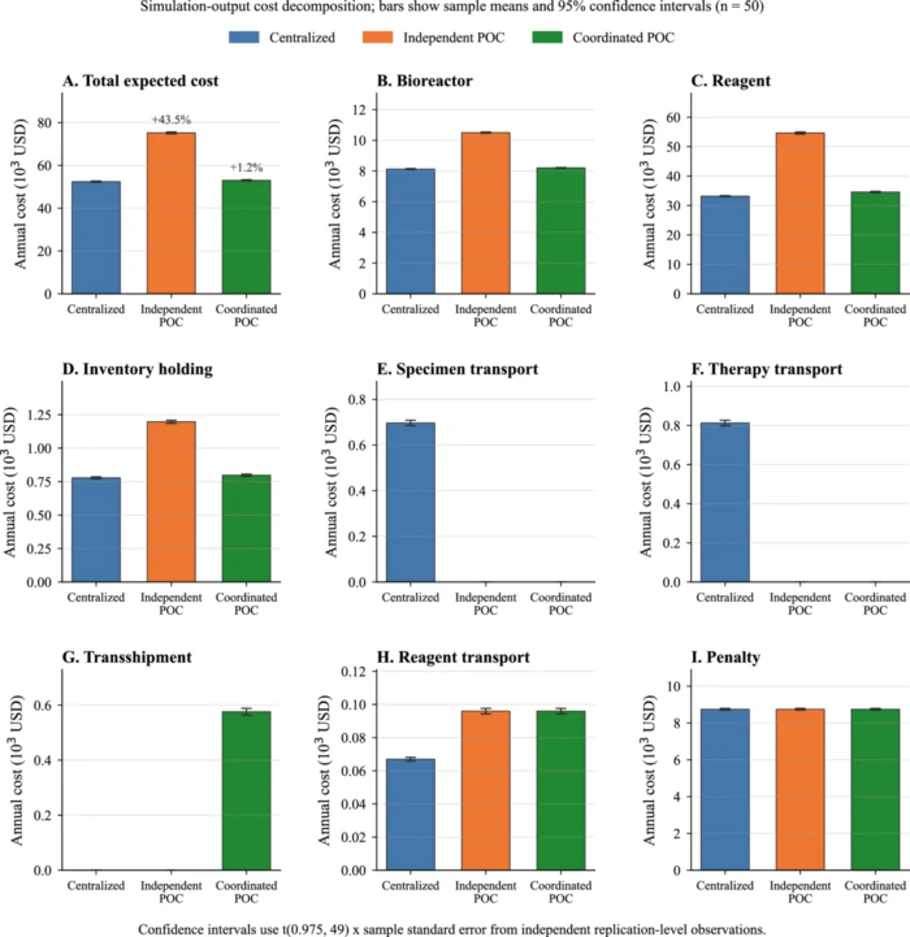
Annual cost decomposition for the centralized, independent POC, and coordinated POC network configurations under the baseline numerical case study. Bars represent sample mean annual costs across R=50 independent simulation replications, and error bars denote two‐sided 95% confidence intervals for the mean computed as $\bar{x}\pm t_{0.975,49}s∕\sqrt{50}$. The part labels denote the following cost components (A) total expected cost, (B) bioreactor cost, (C) reagent cost, (D) inventory holding cost, (E) specimen transportation cost, (F) therapy transportation cost, (G) transshipment cost, (H) reagent transportation cost, and (I) penalty cost.

When replication‐level output is used to quantify simulation uncertainty, the sample standard deviation, standard error, and two‐sided 95% confidence interval are computed as

$$s_{jk}=\sqrt{\frac{1}{R-1}\sum_{r=1}^{R}{\left(C_{rjk}-{\hat{\mu }}_{jk}\right)}^{2}},SE\left({\hat{\mu }}_{jk}\right)=\frac{s_{jk}}{\sqrt{R}},$$

$${\hat{\mu }}_{jk}\pm t_{0.975,R-1}\;SE\left({\hat{\mu }}_{jk}\right).$$

For pairwise comparisons of total expected cost, the same output‐analysis framework can be applied to replication‐level cost differences between network configurations. Rather than treating a fixed number of replications as sufficient by assumption, the adequacy of the replication budget is assessed through the width of the confidence intervals relative to the decision‐relevant cost differences. With R=50, the paired total‐cost comparisons yielded 95% confidence intervals of 22.615 to 22.959 thousand USD for independent POC minus centralized, 0.507 to 0.701 thousand USD for coordinated POC minus centralized, and −22.352 to −22.014 thousand USD for coordinated POC minus independent POC. These intervals are narrow relative to the principal cost differences and support the qualitative ranking of the three network designs. Additional replications would further reduce simulation error, but the reported intervals indicate that the main cost‐comparison conclusions are not driven by replication‐level stochastic noise. This distinction is important for interpretation: Figure 2 is a cost‐decomposition figure whose bars report verified replication‐based sample means, whereas confidence intervals or hypothesis tests require the underlying replication‐level observations and should not be inferred from aggregate means alone.

### 6.3 Cost Comparison

Thus, compared to the centralized network, the independent POC network requires 29% more bioreactors and has a 54% greater total reagent base stock level. However, the coordinated POC network requires only a slight increase in the number of bioreactors and total network base stock level compared to the centralized network, significantly reducing the capital expenditure and inventory holding cost advantage of the centralized network. Notably, this advantage can be reduced to zero (at a modest increase in transshipment cost and total cost) and still satisfy the patient service level.

The transportation cost structure is defined as follows:

- For the centralized network, transportation costs include the cost of transporting reagents from the reagent suppliers to the manufacturing facility, the cost of transporting the specimens from the POCs to the facility, and the cost of transporting the cell therapies from the facility to the POCs.
- For the independent POC network, transportation costs include the cost of transporting reagents from the reagent suppliers to the POCs.
- For the coordinated POC network, transportation costs include the cost of transporting reagents from the reagent suppliers to the POCs. Additionally, (i) when transshipment of specimens is needed, the cost of transporting the transshipped specimens to the POCs that will manufacture the concomitant cell therapies and the cost of transporting the cell therapies back to the home POCs and (ii) when transshipment of reagents is needed, the cost of transporting the transshipped reagents to the POCs that need reagents for cell therapy manufacturing (a form of inventory balancing).

The analysis assumes two supplier locations. Supplier 1, located in San Jose, CA, provides reagents for the POCs on the West Coast, the Colorado Blood Cancer Institute, and the Baylor University Medical Center; Supplier 2, located in Waltham, MA, provides reagents for the remainder of the POCs. For the centralized network, Supplier 1 is assumed to provide 90% of reagents to the manufacturer, and Supplier 2 is assumed to provide 10% of the needed reagents. Although a sensitivity analysis indicates that changes in these percentages have little impact on total expected cost, other transportation costs may be sensitive to changes in the locations of the centralized facility, the POCs, and the reagent suppliers.

Cost performance is evaluated first because economic scalability is a necessary condition for commercial deployment of distributed AuCT manufacturing. For each network design, Table 1 reports the annualized cost components associated with the baseline numerical study. The values are the estimators ${\hat{\mu }}_{jk}$ defined above, reported as sample mean annual costs across 50 independent AnyLogic simulation replications. Capital costs for cryopreservation are assumed to be identical across network designs and are therefore omitted from the comparison. The central managerial result is that the coordinated POC network remains within approximately 1.2% of the total expected cost of the centralized network while avoiding the substantially higher cost of the independent POC network, whose total expected cost is approximately 43.5% higher than the centralized benchmark. The transshipment component in the coordinated POC case is driven by the fact that transshipment occurs for reagents and/or specimens with a probability of 0.193.

**TABLE 1:** Network cost comparison for the centralized, independent POC, and coordinated POC configurations. Values shown are replication‐based sample mean annual costs, ${\hat{\mu }}_{jk}$, across R=50 independent AnyLogic simulation replications.

Cost component	Centralized ($)	Independent POC ($)	Coordinated POC ($)
Bioreactor	8125	10,500	8200
Reagent	33,153	54,627	34,567
Inventory holding	778	1197	798
Specimen transport	697	0	0
Therapy transport	813	0	0
Transshipment	0	0	576
Reagent transport	67	96	96
Penalty	8750	8750	8750
Total expected cost	52,383	75,170	52,987

Figure 2 complements Table 1 by displaying the total cost and the individual cost components on separate panels. This presentation is appropriate for the cost comparison because the main question is not whether a single aggregate cost differs across designs, but which capital, inventory, transportation, transshipment, and penalty components drive the commercial trade‐off. Bars report the replication‐based sample mean annual cost ${\hat{\mu }}_{jk}$ for each cost component and network design. Because the comparison concerns prespecified network designs under stochastic simulation rather than independently sampled clinical or manufacturing groups, Figure 2 is interpreted as a simulation‐output cost‐decomposition summary. Formal uncertainty intervals for Figure 2 should be computed from the retained replication‐level observations using the estimators defined above rather than approximated from aggregate means.

The figure highlights that the independent POC network is mainly penalized by higher bioreactor and reagent costs, which is consistent with the larger installed‐capacity and base‐stock requirements reported above. By contrast, the coordinated POC network adds a transshipment cost component but keeps bioreactor, reagent, and inventory holding costs close to the centralized benchmark. This pattern explains why coordinated POC manufacturing can narrow the cost gap with centralized production while retaining a distributed manufacturing structure.

### 6.4 Turnaround Time Analysis

Turnaround time is defined here as the average time from specimen harvest at the patient's home POC to therapy infusion at that same POC. Holding manufacturing duration fixed, shorter turnaround time represents greater patient benefit because it reduces the interval between specimen collection and treatment availability. Two scenarios are considered:

- **Scenario 1.** Each specimen and therapy are cryopreserved for all cases, adding freezing, thawing, and recovery time(s) to therapy manufacturing time and transportation time(s) to determine the total turnaround time. Cryopreservation is assumed to add 3 days to turnaround time: 1 day for freezing and other preparation processes and 2 days for thawing, pre‐conditioning, packaging, and related activities.
- **Scenario 2.** If therapy manufacturing occurs at the patient's home POC, the specimen and therapy are assumed not to require cryopreservation when ambient technology is used. Otherwise, when transshipment is required, specimen and therapy cryopreservation occur as in the first scenario.

The transportation times between the centralized manufacturing site, the suppliers, and the POCs are given in Appendix A. Table 2 reports the resulting average turnaround times. The table is placed here to make the cost‐responsiveness trade‐off explicit: Table 1 and Figure 2 describe the economic side of the network‐design decision, whereas Table 2 describes the patient‐facing operational side.

**TABLE 2:** Average network turnaround time under different manufacturing configurations.

Network configuration	Avg. turnaround time (days)
Centralized	29.5
Independent POC	27.5
Coordinated POC	28.2
Independent POC (no cryopreservation)	21.5
Coordinated POC with transshipment (cryopreserved)	23.9

The centralized network has the longest turnaround time in the baseline cryopreservation scenario because specimens and therapies must move between POCs and the centralized manufacturing site. The independent POC network has the shortest baseline turnaround time, 27.5 days, but achieves this responsiveness with substantially higher bioreactor and reagent requirements. The coordinated POC network has an intermediate average turnaround time of 28.2 days, only 0.7 days longer than the independent POC network and 1.3 days shorter than the centralized network. This result is important for commercial AuCT deployment because it shows that coordination can preserve much of the responsiveness of local manufacturing while avoiding most of the cost penalty of fully independent POCs.

### 6.5 Cost Implications With Ambient Technology

Ambient technology can further affect the economic interpretation of distributed manufacturing. Under the assumptions used here, yield increases from 1 to 1.15, resulting in a 15% increase in output per unit of raw material. This results in savings in reagent cost per therapy with increased yield of approximately 3913 USD. However, there is an additional cost for ambient transportation per therapy, which is estimated to be 2500 USD.

Considering the savings in reagent cost and the extra transportation cost, the net savings per therapy are 1413 USD. Thus, ambient technology may strengthen the commercial case for distributed manufacturing when the operational benefit of avoiding cryopreservation is paired with a net per‐therapy cost saving under the assumed yield and transportation‐cost values.

### 6.6 Comparison With Previous Work

The results are next compared with those reported in Wang et al. (n.d). It was found in Wang et al. (n.d) that the total expected cost of the centralized network accounted for 67.0% of the cost associated with the independent POC network. The model discussed in this paper calculates this percentage to be 68.3%, demonstrating a strong concordance with the slightly differing model in Wang et al. (n.d) regarding this comparison.

Moreover, (Wang et al. n.d) determined that the total expected cost of the coordinated POC network was 78.0% of the independent POC network's cost. In contrast, the estimate in the present study is 68.7%. Notably, this result is within a 1% margin of the analogous percentage for the centralized network, reinforcing the assertion that the expected total costs for both the coordinated POC network and the centralized network are remarkably similar. The decrease in the coordinated POC network cost found in this study, in comparison to Wang et al. (n.d), is primarily attributable to two elements:

- **Capacity Planning Algorithm:** The deployment of the capacity planning algorithm delineated herein led to a reduced number of bioreactors for the independent POC network, relative to the findings in Wang et al. (n.d).
- **Optimal Transshipment Algorithm:** The heuristic‐based transshipment algorithm featured in Wang et al. (n.d) provided seemingly adequate initial results. In the present study, the transshipment algorithm minimizes costs through an integer program (IP) approach.

There were several other differences in the modeling assumptions made in Wang et al. (n.d) and the present study, including the assumption in this study that labor cost per specimen is equal for both networks whereas the labor cost assumptions made in Wang et al. (n.d) implied that the POC network had a slightly higher labor cost than its centralized counterpart. A preliminary sensitivity analysis supports the claim that these differences have not significantly affected the above numerical results.

## 7 Conclusion

This study compares centralized, coordinated POC, and independent POC AuCT manufacturing networks using an MDP‐based framework that incorporates reagent replenishment, transshipment, patient deterioration, and bioreactor capacity planning. The analysis shows that the coordinated POC network is bounded between the centralized and independent POC designs in terms of expected cost and, for the baseline numerical case study, achieves a total expected cost that is within 1% of the centralized network while remaining substantially less costly than the independent POC network.

From the standpoint of patient benefit, the coordinated POC network also preserves much of the turnaround‐time advantage of distributed manufacturing. In the baseline cryopreservation scenario, the coordinated POC network has an average turnaround time of 28.2 days, compared with 29.5 days for the centralized network and 27.5 days for the independent POC network. Thus, the coordinated POC network reduces average turnaround time by 1.3 days relative to the centralized network while remaining only 0.7 days longer than the independent POC network. When cryopreservation can be avoided for therapies produced at the home POC, the independent POC and coordinated POC networks have average turnaround times of 21.5 and 23.9 days, respectively, further highlighting the patient‐access benefit of distributed manufacturing.

Taken together, these findings support the view that coordinated POC manufacturing is an important AuCT network design option for commercialization and industrialization. It can retain much of the economic efficiency of centralized production while preserving most of the responsiveness associated with local manufacturing. In practical terms, this means that coordinated POC manufacturing may provide manufacturers with a way to limit the capital and inventory burden associated with fully independent local manufacturing, while still offering treatment centers and patients shorter effective turnaround times than a purely centralized system.

The managerial implications differ by stakeholder. For manufacturers, the results suggest that coordinated distributed manufacturing may reduce the need to choose strictly between low‐cost centralized production and high‐responsiveness independent local production. For POCs and treatment centers, the coordinated design highlights the operational value of shared visibility, transshipment capability, and common decision rules when local demand and resource availability vary across sites. For patients, the relevant implication is not a direct clinical outcome claim, but an operational proxy for patient benefit: shorter time from specimen harvest to therapy infusion may support faster treatment availability under the assumptions of the model. For supply‐chain planners, the comparison clarifies which resources must be coordinated at commercial scale: installed bioreactor capacity, reagent base stock, cross‐site movement of specimens and reagents, and transportation links between suppliers, manufacturing sites, and POCs. These implications align with prior arguments that decentralized CGT manufacturing requires not only local manufacturing capability, but also robust coordination, quality governance, and delivery infrastructure (Harrison et al. 2018; Elverum and Whitman 2020).

The contribution of the present study is therefore twofold. First, it provides an analytic framework for comparing alternative AuCT manufacturing network designs under a common set of operational assumptions. Second, it offers managerial insight into how coordination, transshipment, and capacity planning influence the balance between cost efficiency and patient responsiveness in multi‐site AuCT manufacturing systems. These results are relevant to manufacturers, treatment centers, and supply‐chain planners evaluating how to configure future CGT manufacturing networks, particularly as the field moves toward larger‐scale deployment.

Several important questions remain outside the scope of the present analysis. The study does not explicitly address broader regulatory implementation, cross‐site quality consistency, or large‐scale sensitivity analysis across all stochastic and cost parameters. In addition, the feasibility of sustained network‐wide coordination in real clinical settings may depend on digital infrastructure, governance, and operational visibility that are not modeled here. Future work should therefore examine the impact of network design on resilience, the effect of perishability on replenishment and transshipment policies, and the robustness of the conclusions under alternative demand, service‐level, and discount‐factor assumptions.

## Author Contributions

**Zhaowei Li:** conceptualization, methodology, formal analysis, software, investigation, writing – original draft. **Chelsea C. White III:** conceptualization, methodology, supervision, writing – review and editing. **Kan Wang:** data curation, validation, writing – review and editing. **Ben Wang:** conceptualization, supervision, writing – review and editing.

## Ethics Statement

The authors have nothing to report.

## Consent

The authors have nothing to report.

## Conflicts of Interest

The authors declare no conflicts of interest.

## Data Availability Statement

The data that support the findings of this study are available from the corresponding author upon reasonable request.

## Appendix A Transportation Distance, Time, and Price to Manufacturing Sites

Table A1

**TABLE A1:** Numbered POC key for Figure 1.

No.	POC/Treatment Center	No.	POC/Treatment Center
1	Seattle Cancer Care Alliance	11	Norton Cancer Institute
2	Oregon Health and Science University Hospital	12	Cancer Treatment Centers of America Chicago
3	Stanford Health Care	13	Massachusetts General Hospital Cancer Center
4	UCLA Health	14	Memorial Sloan Kettering Cancer Center
5	UC San Diego Health	15	Penn State Cancer Institute
6	Mayo Clinic Arizona	16	MedStar Georgetown University Hospital
7	Colorado Blood Cancer Institute	17	Levine Cancer Institute
8	Baylor University Medical Center/Texas Oncology	18	Winship Cancer Institute of Emory University
9	Mayo Clinic	19	Mayo Clinic Florida
10	Siteman Cancer Center	20	Miami Cancer Institute

Table A2

**TABLE A2:** Transportation distance, time, and price to manufacturing sites.

Function	Organization	State	Address	Distance to CA manufacturing site (mile)	Duration to CA manufacturing site	Cost to CA manufacturing site (USD)
Manufacturing	Kite Pharma	California	2355 Utah Ave, El Segundo, CA 90245 USA	0		497
Treatment Center	Seattle Cancer Care Alliance	Washington	825 Eastlake Ave E, Seattle, WA 98109	1200	2 h 50 min	312
Treatment Center	Oregon Health and Science University Hospital	Oregon	3181 SW Sam Jackson Park Rd, Portland, OR 97239	1000	2 h 20 min	287
Treatment Center	Stanford Health Care	California	211 Quarry Rd #302, Palo Alto, CA 94304	400	1 h 15 min	97
Treatment Center	UCLA Health	California	700 W 7th St S270‐D, Los Angeles, CA 90017	20	35 min	0
Treatment Center	UC San Diego Health	California	200 W Arbor Dr, San Diego, CA 92103	120	2 h 8 min	0
Treatment Center	Mayo Clinic Arizona	Arizona	5701 E Mayo Blvd, Phoenix, AZ 85054	485	1 h 15 min	281
Treatment Center	Baylor University Medical Center/Texas Oncology ‐ Baylor Charles A. Sammons Cancer Center	Texas	3410 Worth St, Suite 400, Dallas, TX 75246	1500	2 h 50 min	285
Treatment Center	Colorado Blood Cancer Institute in partnership with Sarah Cannon Cancer Institute at Presbyterian/St. Luke's Medical Center	Colorado	1721 E 19th Ave #300, Denver, CO 80218	1100	2 h 15 min	252
Treatment Center	Mayo Clinic	Minnesota	200 1st St SW, Rochester, MN 55905	2000	5 h 30 min	374
Treatment Center	Siteman Cancer Center	Missouri	4921 Parkview Pl, St. Louis, MO 63110	1900	3 h 25 min	464
Treatment Center	Norton Cancer Institute	Kentucky	4950 Norton Healthcare Blvd, Suite 300, Louisville, KY 40241	2200	4 h 5 min	352
Treatment Center	Cancer Treatment Centers of America Chicago	Illinois	2520 Elisha Ave, Zion, IL 60099	2100	3 h 50 min	314
Treatment Center	Massachusetts General Hospital Cancer Center	Massachusetts	55 Fruit St, Boston, MA 02114	3000	5 h 20 min	506
Treatment Center	Memorial Sloan Kettering Cancer Center	New York	1275 York Ave, New York, NY 10065	2900	5 h 10 min	625
Treatment Center	Penn State Cancer Institute	Pennsylvania	400 University Dr, Hershey, PA 17033	2700	6 h 40 min	672
Treatment Center	MedStar Georgetown University Hospital	Washington, DC	3800 Reservoir Rd NW, Washington, DC 20007	2700	4 h 45 min	497
Treatment Center	Levine Cancer Institute	North Carolina	1021 Morehead Medical Dr, Charlotte, NC 28204	2500	4 h 30 min	428
Treatment Center	Winship Cancer Institute of Emory University	Georgia	1365 E Clifton Rd NE Building C, Atlanta, GA 30322	2200	4 h	377
Treatment Center	Mayo Clinic Florida	Florida	4500 San Pablo Rd S, Jacksonville, FL 32224	2500	4 h 25 min	588
Treatment Center	Miami Cancer Institute	Florida	8900 N Kendall Dr, Miami, FL 33176	2800	4 h 40 min	570

## Appendix B Cost Data in USD

Table B1

**TABLE B1:** Cost data in USD.

Resource type	Resource name	Cost per year/USD	Cost per hour/USD	Cost per use/USD	Cost per day/USD	Cost per item/USD
Consumables	Liquid nitrogen			10		
Consumables	Liquid nitrogen		0.5			
Consumables	Sepax consumables kit			100		
Consumables	Mycoplasma detection kit					31.55
Consumables	CliniMACS reagent					4000
Consumables	Leukapheresis kit					2000
Consumables	Expansion media				28	
Consumables	Transduction kit					1070
Consumables	T‐cell activation kit					645
Consumables	Prodigy kit					4630
Consumables	Aseptic gowning					7.5
Equipment	Shipping container	63.5				
Equipment	Sepax					7999
Equipment	CliniMACS Prodigy	20,000				
Equipment	Barcode reader	120				
Equipment	Peltier block	180				
Equipment	Microscope	1100				
Equipment	Vi‐Cell	6550				
Equipment	Flow cytometer	6550				
Equipment	PCR	5898				
Equipment	ELISA plate reader	5000				
Equipment	Two‐glove isolator	20,000–56,008				
Equipment	Incubator	2300				
Equipment	Endosafe	1214.5				
Equipment	BacT/Alert	3250				
Equipment	Refrigerator	720				
Equipment	BacT/Alert	3250				
Equipment	Refrigerator	720				
Equipment	Barcode reader					6000–10,000
Equipment	Freezer (−20)					6000–10,000
Equipment	Freezer (−80)	720				
Equipment	Peltier block	180				
Equipment	Two‐glove isolator	23,834				
Equipment	CRF (facility)	1600				
Equipment	Cryogenic freezer					2000–20,000
Equipment	Autoclave	28,000				
Facility	Annual revalidation	25,000				
Facility	Utility cost (per m2)		0.00588			
Facility	EMS, handheld portable particle counter	300				
Facility	EMS, annual calibration and maintenance cost	5000				
Hospital	Operating theater cost		1562.03			
Hospital	Emergency and intensive care		125			
Hospital	Lymphodepletion jabs			1562.03		
Personnel	Nurses		30.24			
Personnel	Chief surgeon		167.92			
Personnel	Pharmacist		33.3			
Personnel	Logistics technicians		25			
Personnel	Clinical support worker		28.64			
Personnel	QC personnel	35,000				
Personnel	Production personnel	35,000				
Personnel	QP	80,000				
Personnel	QA personnel	35,000				
Transport	Transportation cost		20			
